# Author Correction: Plants interfere with non-self recognition of a phytopathogenic fungus via proline accumulation to facilitate mycovirus transmission

**DOI:** 10.1038/s41467-025-62687-w

**Published:** 2025-08-11

**Authors:** Du Hai, Jincang Li, Daohong Jiang, Jiasen Cheng, Yanping Fu, Xueqiong Xiao, Huanran Yin, Yang Lin, Tao Chen, Bo Li, Xiao Yu, Qing Cai, Wei Chen, Ioly Kotta-Loizou, Jiatao Xie

**Affiliations:** 1 National Key Laboratory of Agricultural Microbiology, Huazhong Agricultural University, Wuhan, Hubei China; 2 The Provincial Key Lab of Plant Pathology of Hubei Province, College of Plant Science and Technology, Huazhong Agricultural University, Wuhan, Hubei China; 3 Hubei Hongshan Laboratory, Wuhan, Hubei China; 4 National Key Laboratory of Crop Genetic Improvement and National Center of Plant Gene Research (Wuhan), Huazhong Agricultural University, Wuhan, China; 5 School of Life and Medical Sciences, University of Hertfordshire, Hatfield, UK; 6 Department of Life Sciences, Imperial College London, London, UK

**Keywords:** Viral transmission, Microbial ecology, Fungal ecology

Correction to: *Nature Communications* 10.1038/s41467-024-49110-6, published online 04 June 2024

The original version of this Article contained errors in Figure caption 4 A and citations in the discussion section.

In the caption to Fig. 4 A the used fungal strain was incorrectly referenced as Ep-1PNA367G.

This has been amended to strain SCH733.

In the discussion section references in the following sentence have been incorrectly cited: “For example, the transmission rate of the prototypic CHV1 between incompatible strains of C. parasitica on trees consistently surpasses that observed in in vitro experiments [40,41], and mycovirus transmission can occur between incompatible R. necatrix strains on apple branches [42,43].“

This has been amended to the correct citations [28] and [29], respectively.

These errors have now been corrected in the PDF and HTML versions of the Article.

