# Supplementary material for: Plants interfere with non-self recognition of a phytopathogenic fungus via proline accumulation to facilitate mycovirus transmission
Source: Nat Commun. 2024 Jun 4;15:4748. doi: 10.1038/s41467-024-49110-6 (PMC11150657; doi:10.1038/s41467-024-49110-6)
Supplement: Supplementary file 1 — Supplementary Information [file 41467_2024_49110_MOESM1_ESM.pdf]

Supplementary Information for

Plants interfere with non-self recognition of a phytopathogenic fungus *via* proline accumulation to facilitate mycovirus transmission

Du Hai[1,2,3], Jincang Li[1,2,3], Daohong Jiang[1,2,3], Jiasen Cheng[1,2], Yanping Fu[1,2], Xueqiong Xiao[1,2], Huanran Yin[4], Yang Lin[1,2], Tao Chen[1,2,3], Bo Li[1,2,3], Xiao Yu[1,2,3], Qing Cai[1], Wei Chen[4], Ioly Kotta-Loizou[5,6], Jiatao Xie[1,2,3]*

Supplementary materials for this manuscript include the following:

Supplementary Fig. 1-12

Supplementary Table 1 and 2

Supplementary Fig.1

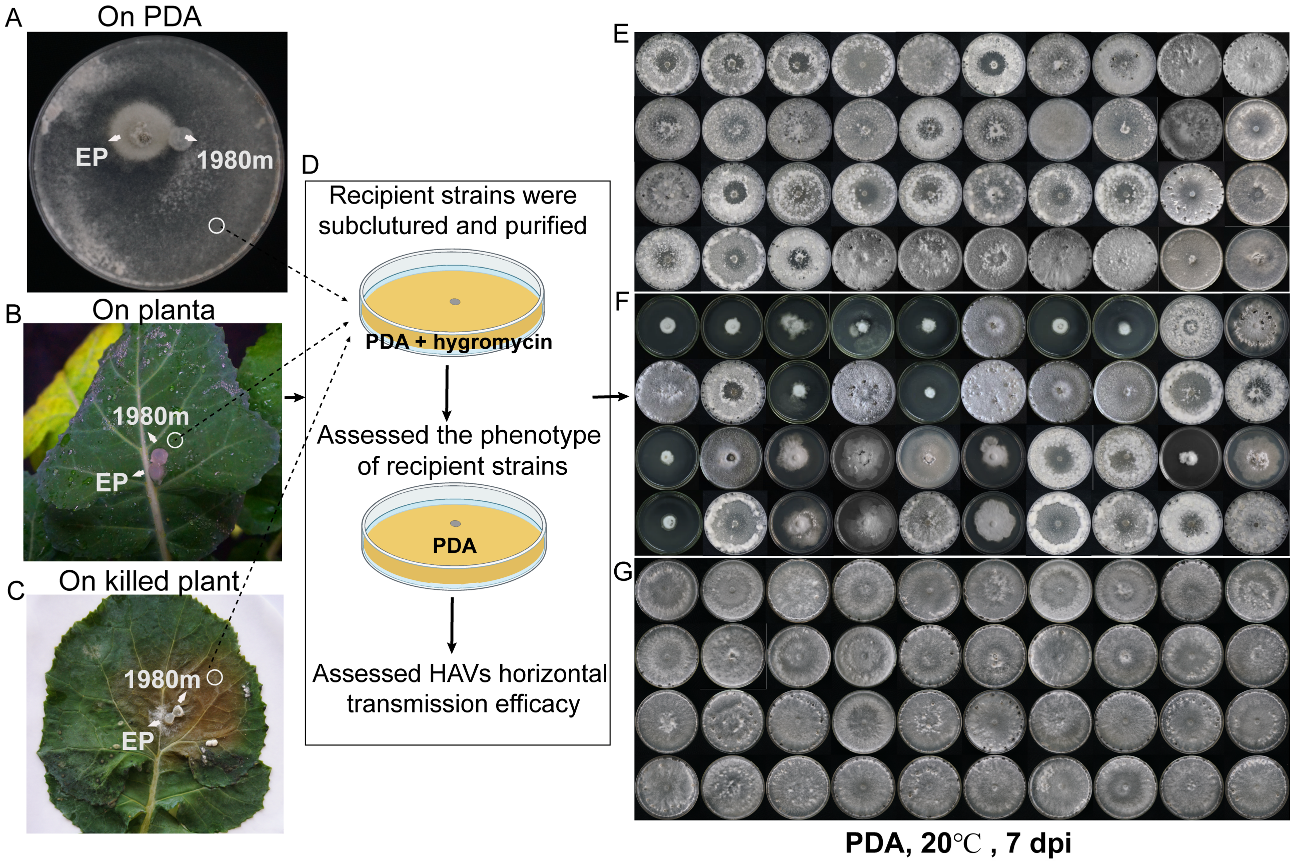

Supplementary Fig. 1 Oilseed rape plants enhance mycovirus transmission.

Strain Ep-1PNA367 was dual-cultured with strain 1980m on PDA (A), and on the living oilseed rape leaves (B) and cold (-80℃)-inactivated oilseed rape leaves (C) for 3 days. (D) The diagram for re-isolation and subculture of isolates picked up from strain 1980m side. The isolates were obtained by re-isolating from the strain 1980m side after dual-cultured with Ep-1PN. Subsequently, they were subcultured on PDA containing hygromycin to eliminate the influence of the donor strain, ensuring the retention of only recipient strains with hygromycin resistance. (E-G) Then re-isolates were cultured on PDA for 7 days to observe their colony morphology. (D), (E), and (F) represent the colony morphology of re-isolates picked up from stain 1980m side of (A), (B) and (C). Most of the re-isolates from strain 1980m that co-inoculated with strain Ep-1PN on the living oilseed

rape leaves show abnormal colony morphology (F), but be rarely observed the abnormal colony morphology in the other two treatment groups (E and G).

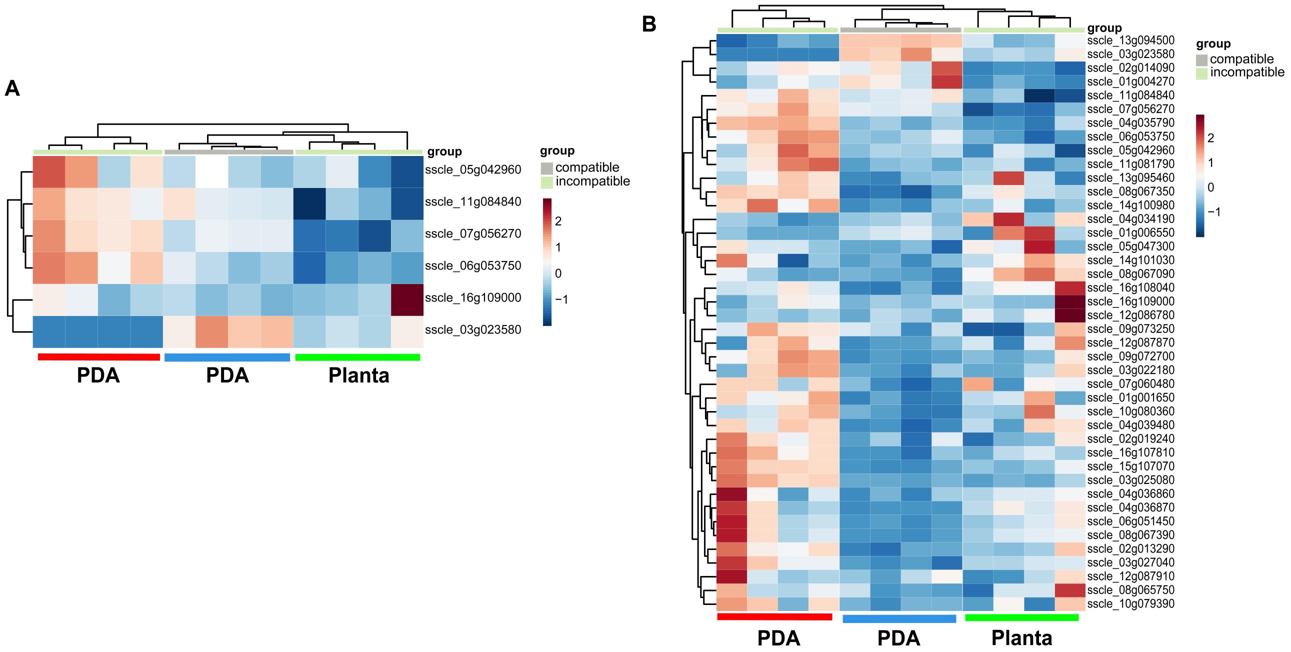

Supplementary Fig. 2 Plants suppress the expression of genes encoding G proteins and Het domain-contained proteins during co-inoculation of two VIC individuals (strains Ep-1PNA367 and 1980) of *S. sclerotiorum*. (A) Expression cluster analysis of genes encoding G protein subunit of *S. sclerotiorum* when the hyphae of strains 1980m contacts with that of Ep-1PNA367 (incompatible) or 1980 (compatible) on PDA and oilseed rape plants (Planta). (B) Expression cluster analysis of the candidate *het* genes that encode proteins containing HET conserved domains in *S. sclerotiorum*, when the hyhae of strains 1980m contacts with that of Ep-1PNA367 (incompatible) or 1980 (compatible) on PDA and oilseed rape plants (Planta). The relative expression of G protein subunit genes and *het* genes was analyzed based on the threshold of RPKM value. Red means a high expression level, and blue indicates a low expression level.

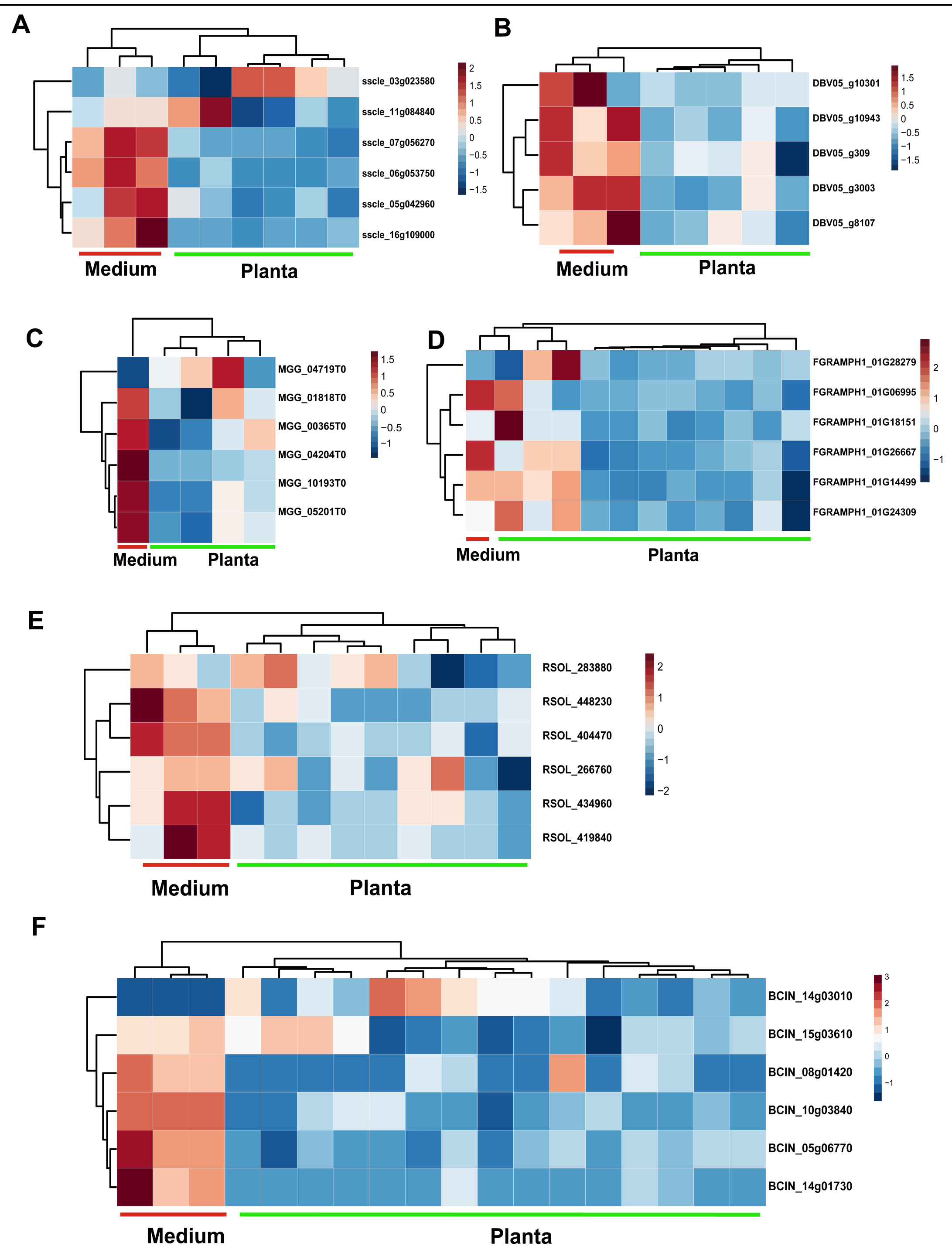

Supplementary Fig. 3 Plants suppress G protein subunit genes expression of the diverse phytopathogenic fungi. Expression cluster analysis of G protein subunit genes in the infection (planta) and vegetative growth (artificial medium) stages of phytopathogenic fungi based on the public transcriptome data. (A), *S. sclerotiorum* infects two varieties of oilseed rape plants (Bioproject Accession: PRJNA516496, 72 hpi); (B) *Lasiodiplodia theobromae* CSS-01s infects

Vitis Vinifera ‘Summer Black’ (Bioproject Accession: PRJNA516496, 8 hpi， 12 hpi, 48 hpi); (C), *Magnaporthe oryzae* infects rice (Bioproject Accession: PRJNA126829, 72 hpi); (D) *Fusarium graminearum* infects wheat (Bioproject Accession: PRJNA486288); (E) *Botrytis cinerea* infects tomato (Bioproject Accession: PRJNA505650, 24 hpi); (F) *Rhizoctonia solani* infects potato (Bioproject Accession: PRJEB28197, 72 and 192 hpi). Red means a high expression level, and blue indicates a low expression level.

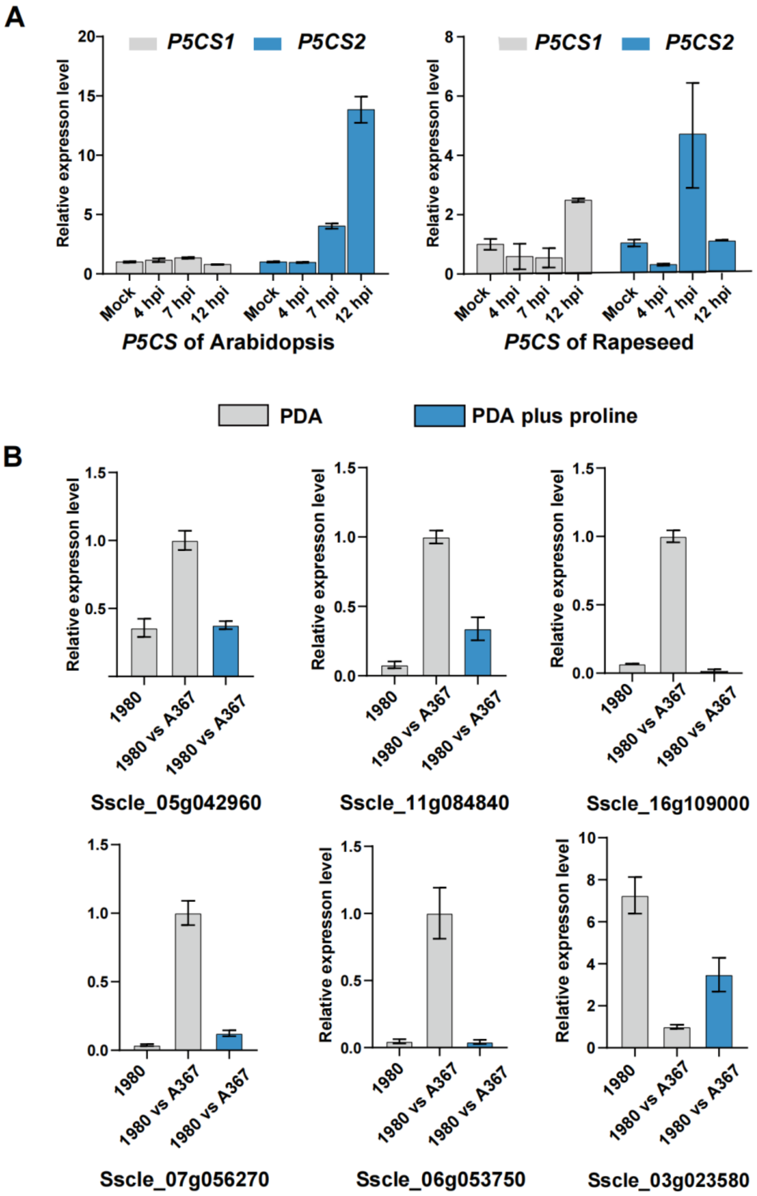

Supplementary Fig. 4 The gene expression profile of proline biosynthesis-related genes, *p5cs1* and *p5cs2*, of the plant upon *S. sclerotiorum* infection, and proline inhibits the expression of G protein subunit genes. (A) qRT-PCR analysis of expression level of proline biosynthesis-related genes *p5cs1* and *p5cs2* of *Arabidopsis* Col-0 and oilseed rape upon *S. sclerotiorum* strain Ep-1PNA367 infection. (B) qRT-PCR analysis of expression level of six G protein subunit genes in strain 1980 alone, and dual-cultured with Ep-1PN on PDA alone and on PDA plus proline. Data are mean ± SD of $n = 3$ independent experiments. Source data are provided as a Source Data file

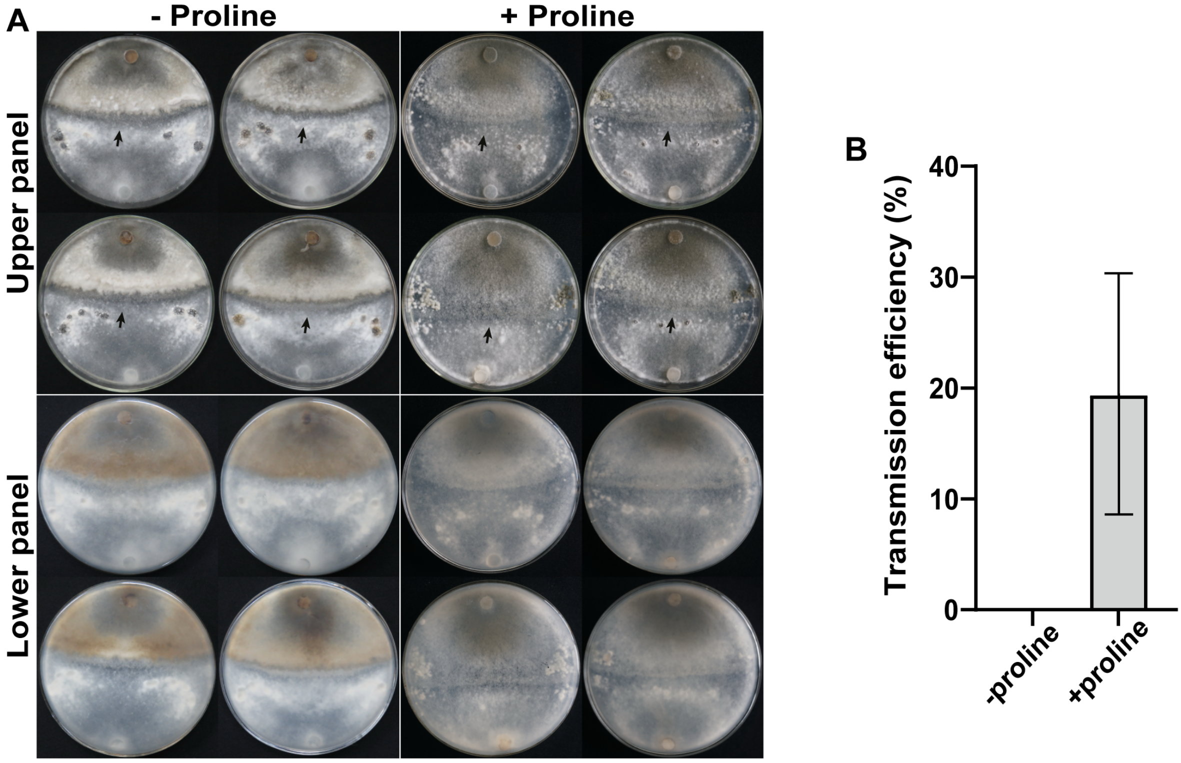

Supplementary Fig. 5 Proline promotes mycovirus transmission from strain SCH733 to strain 1980m. (A) the necrosis zone (arrow) was observed when two incompatible strains SCH733 and 1980m were dual-cultured on PDA in their interface region (-proline), but decrease significantly on PDA containing 3.2 mM proline (+proline). Upper panel, front of culture dish; Lower panel, reverse of cultrue dish. (B) The transmission efficiency of Sclerotinia sclerotiorum narnavirus 5 (SsNaV5) that is potentially related to hypovirulence on strain SCH733 was significantly increased on PDA containing 3.2 mM proline. The information of SCH733 was listed on Supplementary Table S1. Data are mean ± SD of *n* = 20 independent experiments.

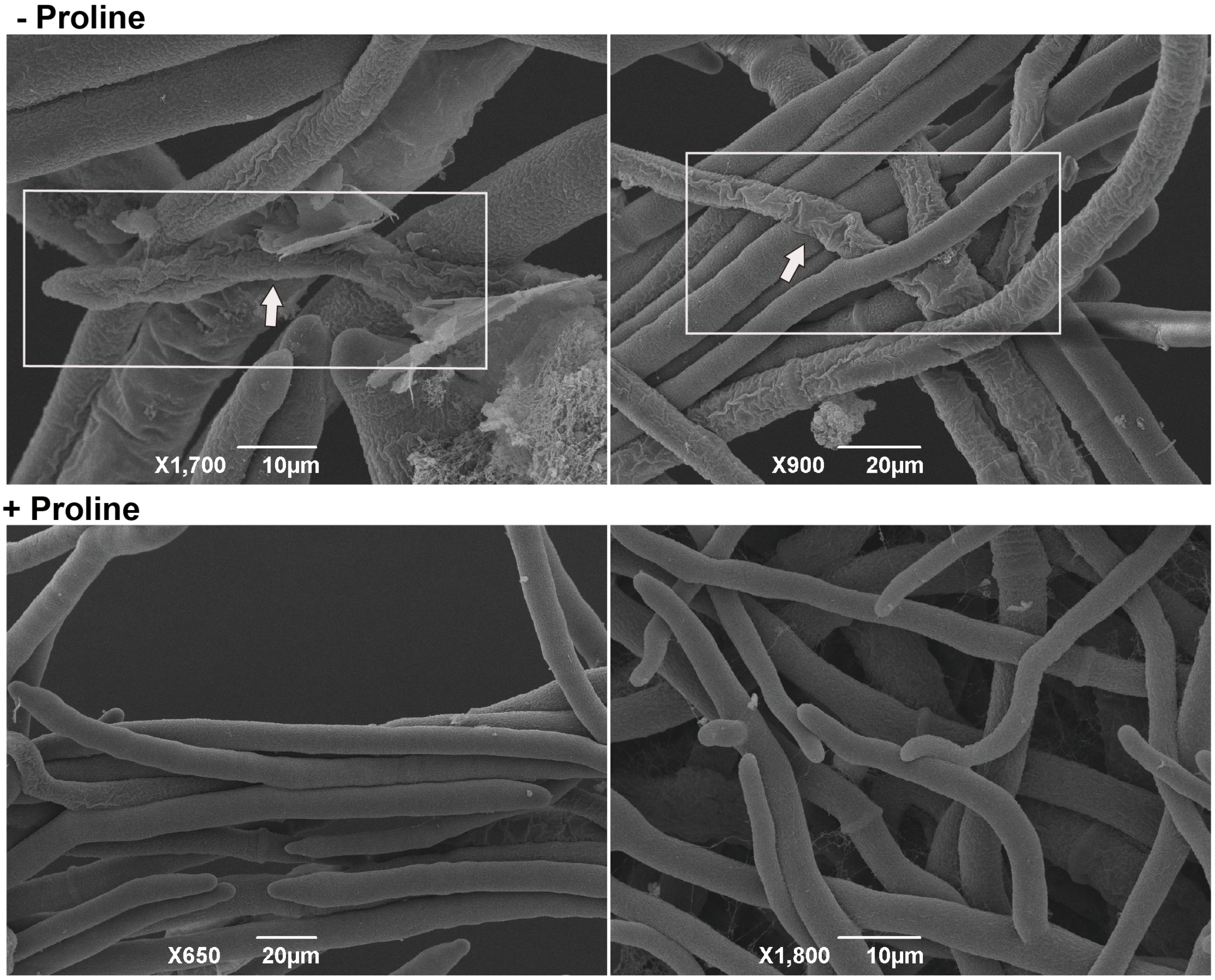

Supplementary Fig. 6 SEM observation of the interaction zone between two incompatible interaction of *S. sclerotiorum* strains 1980m and Ep-1PNA367G. The hyphae degenerate or apoptosis (white arrow) was observed on PDA (upper panel, -proline), but this phenomenon was disappear when proline (3.2 mM) was supplemented on PDA (lower panel, +proline).

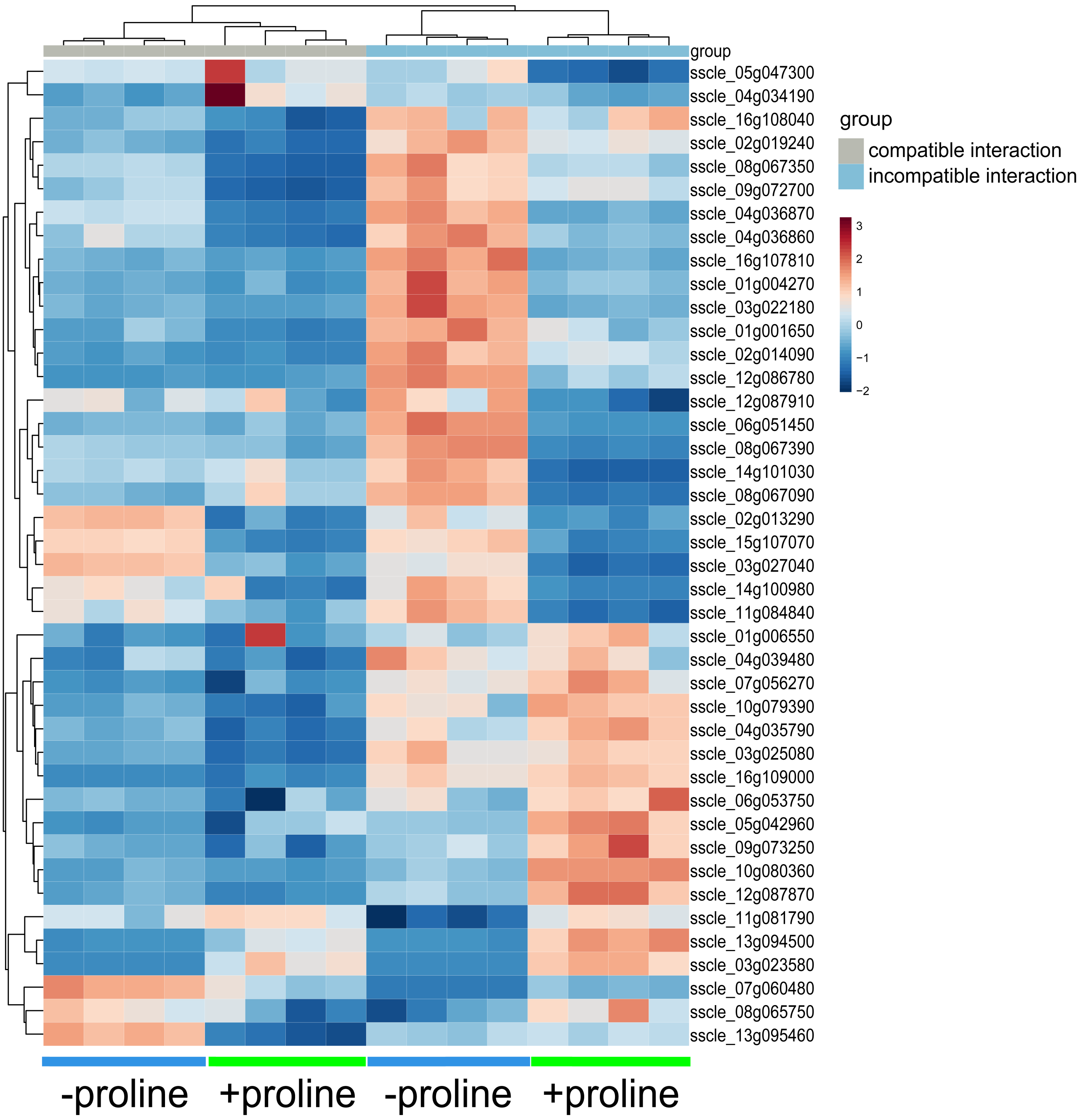

Supplementary Fig. 7 Proline suppresses *vic*-related genes expression during two VIC individuals interaction. Expression cluster analysis of the candidate *het* genes that encode proteins containing HET conserved domains in *S. sclerotiorum* when strains 1980m and Ep-1PNA367 (incompatible) or 1980m (compatible) hyphae interacts on PDA with proline (+proline) or on PDA without proline (-proline). The relative expression of *vic*-related genes were analyzed

based on the threshold of RPKM value. Red means a high expression level, and blue indicates a low expression level.

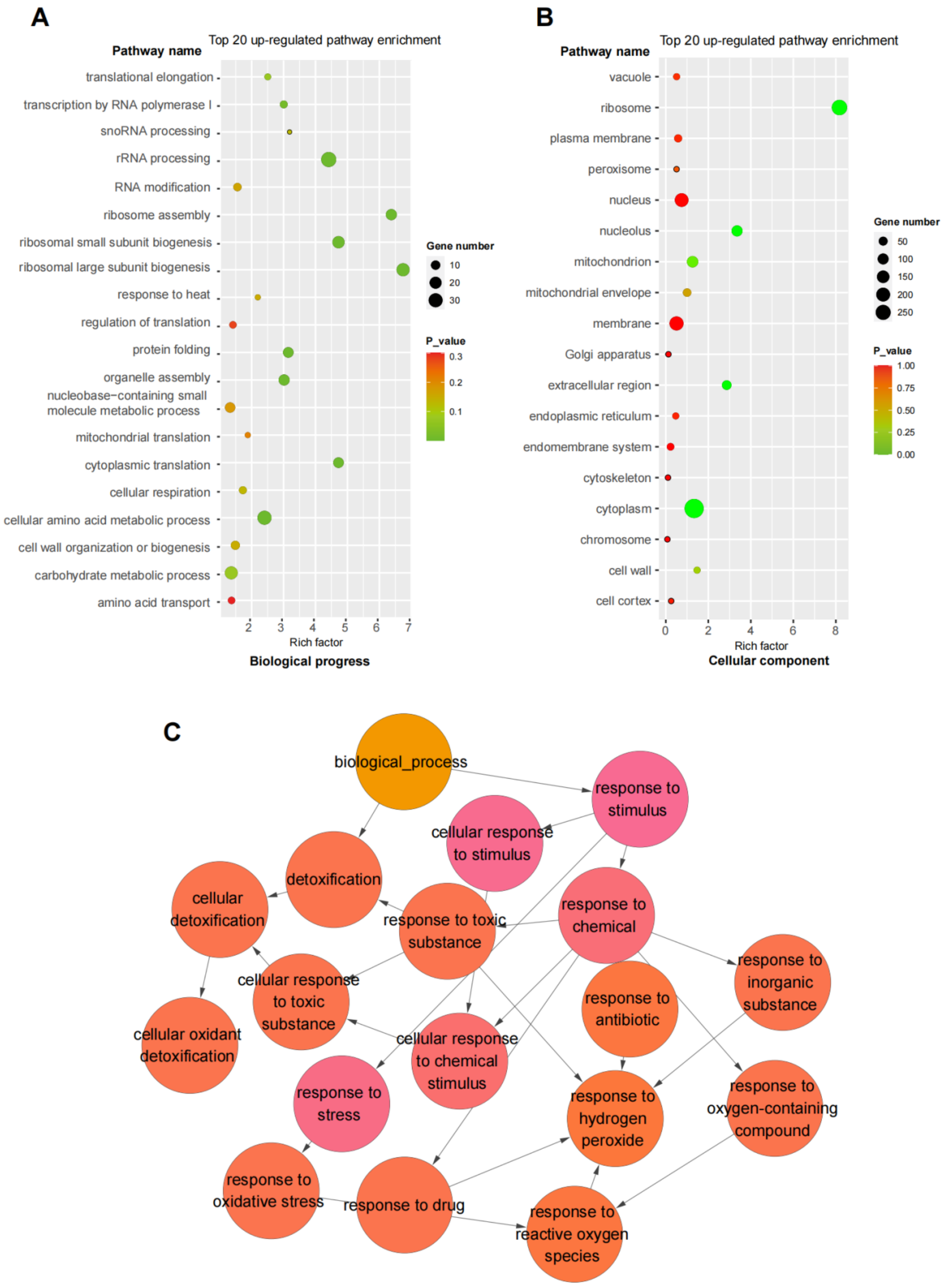

Supplementary Fig. 8 GO enrichment analysis of the up-regulated DEGs with fold change >2 in two incompatible interaction of *S. sclerotiorum* strains 1980m and Ep-1PNA367G that was induced by proline (3.2 mM). The top 20 most significantly enriched biological progress (A) and cellular

component (B) based on the results of up-regulated DEGs. (C) Enrichment network analysis of the ROS-related genes that was induced to the down-regulated change by proline.

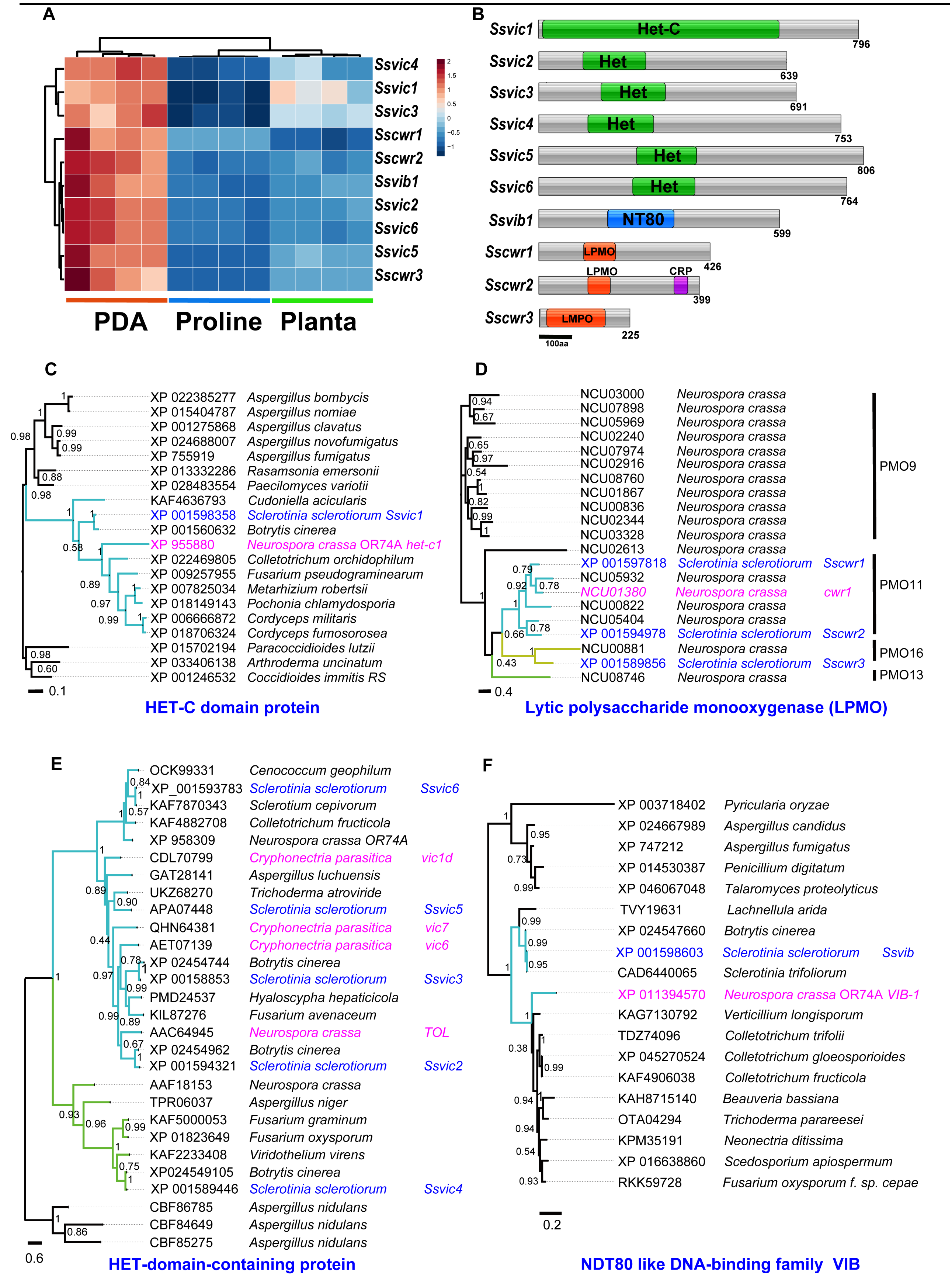

A
Ssvic4
Ssvic1
Ssvic3
Sscwr1
Sscwr2
Ssvib1
Ssvic2
Ssvic6
Ssvic5
Sscwr3
2
1.5
1
0.5
0
−0.5
−1
PDA
Proline
Planta
B
Ssvic1 Het-C 796
Ssvic2 Het 639
Ssvic3 Het 691
Ssvic4 Het 753
Ssvic5 Het 806
Ssvic6 Het 764
Ssvib1 NT80 599
Sscwr1 LPMO 426
Sscwr2 LPMO CRP 399
Sscwr3 LMPO 225
100aa
C
XP 022385277 Aspergillus bombycis
XP 015404787 Aspergillus nomiae
XP 001275868 Aspergillus clavatus
XP 024688007 Aspergillus novofumigatus
XP 755919 Aspergillus fumigatus
XP 013332286 Rasamsonia emersonii
XP 028483554 Paecilomyces variotii
KAF4636793 Cudoniella acicularis
XP 001598358 Sclerotinia sclerotiorum Ssvic1
XP 001560632 Botrytis cinerea
XP 955880 Neurospora crassa OR74A het-c1
XP 022469805 Colletotrichum orchidophilum
XP 009257955 Fusarium pseudograminearum
XP 007825034 Metarhizium robertsii
XP 018149143 Pochonia chlamydosporia
XP 006666872 Cordyceps militaris
XP 018706324 Cordyceps fumosorosea
XP 015702194 Paracoccidioides lutzii
XP 033406138 Arthroderma uncinatum
XP 001246532 Coccidioides immitis RS
0.1
HET-C domain protein
D
NCU03000 Neurospora crassa
NCU07898 Neurospora crassa
NCU05969 Neurospora crassa
NCU02240 Neurospora crassa
NCU07974 Neurospora crassa
NCU02916 Neurospora crassa
NCU08760 Neurospora crassa
NCU01867 Neurospora crassa
NCU00836 Neurospora crassa
NCU02344 Neurospora crassa
NCU03328 Neurospora crassa
NCU02613 Neurospora crassa
PMO9
XP 001597818 Sclerotinia sclerotiorum Sscwr1
NCU05932 Neurospora crassa
NCU01380 Neurospora crassa cwr1
NCU00822 Neurospora crassa
NCU05404 Neurospora crassa
XP 001594978 Sclerotinia sclerotiorum Sscwr2
PMO11
NCU00881 Neurospora crassa
PMO16
XP 001589856 Sclerotinia sclerotiorum Sscwr3
NCU08746 Neurospora crassa
PMO13
0.4
Lytic polysaccharide monooxygenase (LPMO)
E
OCK99331 Cenococcum geophilum
XP_001593783 Sclerotinia sclerotiorum Ssvic6
KAF7870343 Sclerotium cepivorum
KAF4882708 Colletotrichum fructicola
XP 958309 Neurospora crassa OR74A
CDL70799 Cryphonectria parasitica vic1d
GAT28141 Aspergillus luchuensis
UKZ68270 Trichoderma atroviride
APA07448 Sclerotinia sclerotiorum Ssvic5
QHN64381 Cryphonectria parasitica vic7
AET07139 Cryphonectria parasitica vic6
XP 02454744 Botrytis cinerea
XP 00158853 Sclerotinia sclerotiorum Ssvic3
PMD24537 Hyaloscypha hepaticicola
KIL87276 Fusarium avenaceum
AAC64945 Neurospora crassa TOL
XP 02454962 Botrytis cinerea
XP 001594321 Sclerotinia sclerotiorum Ssvic2
AAF18153 Neurospora crassa
TPR06037 Aspergillus niger
KAF5000053 Fusarium graminum
XP 01823649 Fusarium oxysporum
KAF2233408 Viridothelium virens
XP024549105 Botrytis cinerea
XP 001589446 Sclerotinia sclerotiorum Ssvic4
CBF86785 Aspergillus nidulans
CBF84649 Aspergillus nidulans
CBF85275 Aspergillus nidulans
0.6
HET-domain-containing protein
F
XP 003718402 Pyricularia oryzae
XP 024667989 Aspergillus candidus
XP 747212 Aspergillus fumigatus
XP 014530387 Penicillium digitatum
XP 046067048 Talaromyces proteolyticus
TVY19631 Lachnellula arida
XP 024547660 Botrytis cinerea
XP 001598603 Sclerotinia sclerotiorum Ssvib
CAD6440065 Sclerotinia trifoliorum
XP 011394570 Neurospora crassa OR74A VIB-1
KAG7130792 Verticillium longisporum
TDZ74096 Colletotrichum trifolii
XP 045270524 Colletotrichum gloeosporioides
KAF4906038 Colletotrichum fructicola
KAH8715140 Beauveria bassiana
OTA04294 Trichoderma parareesei
KPM35191 Neonectria ditissima
XP 016638860 Scedosporium apiospermum
RKK59728 Fusarium oxysporum f. sp. cepae
0.2
NDT80 like DNA-binding family VIB

Supplementary Fig. 9 (A) The heat map reveals that the expression of ten *S. sclerotiorum* genes was significantly down-regulated when two incompatible strains 1980m and Ep-1PNA367 were co-inoculated on oilseed rape plants (Planta) and PDA containing 3.2 mM proline (Proline), compared that they were dual-cultured on PDA (PDA). (B) Schematic diagram of proteins encoded by ten genes in *S. sclerotiorum*. The Het-related conserved domains were shown with the green frame. The HET-related NDT80 domain were shown with the blue frame, and the cell wall remodeling (LPMO) and chitin recognition protein (CRP) related conserved domains were shown with the orange and purple frames. (C-F) The phylogenetic analysis of proteins encoded by ten genes *Ssvic1* (C), and *Ssvic2*, *Ssvic3*, *Ssvic4*, *Ssvic5*, *Ssvic6* (D), and *Sscwr1*, *Sscwr1*, *Sscwr1* (E), and *Ssvib1* (F), and other related protein in ascomycete fungi. Amino acid sequences of these *vic*-related protein sequences were aligned by employing E-INS-I settings using MAFFT software (version 2.0). The maximum likelihood approach was implemented to construct phylogenetic trees using PhyML 3.0, and branch support was accessed using SPR (Subtree pruning and rafting) branch-swapping. Numbers on the branches indicate the percentage of bootstrap support from 1000 replicates.

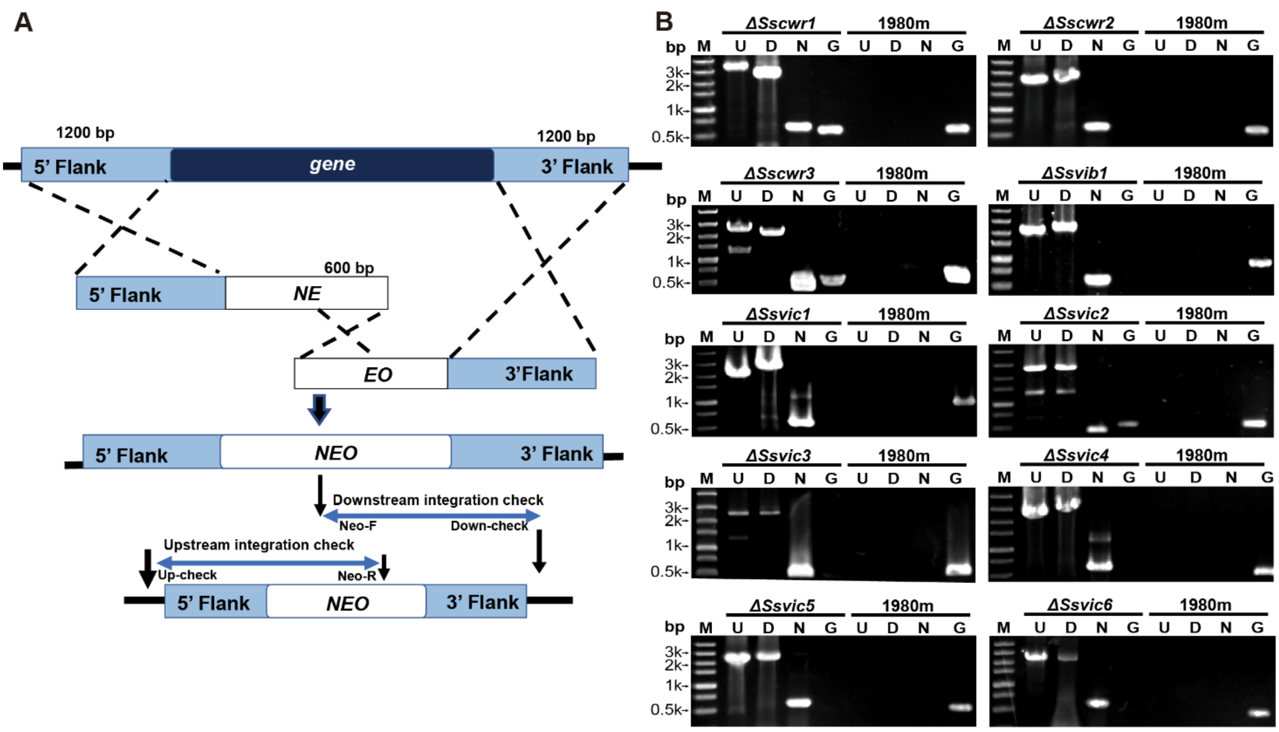

Supplementary Fig. 10 Construction and analysis of *ΔSsvic1* to *ΔSsvic6*, and *ΔSsvib1*, *ΔSscwr1*, *ΔSscwr2*, *ΔSscwr3* deletion mutants. (A) Schematic diagram of targeted gene replacement strategy, the different shading shows the neomycin resistance gene (G418, white box), deletion genes (dark blue), and flanked sequences (light bule box). The scheme was drawn to show gene knock-out strategy using the split marking method. (B) PCR validation of the deletion mutants. Partial G418 gene fragment was amplified with primers NE/EO; the upstream of the targeted deletion genes overlapped trpC promoter was amplified with primers Up-check/Neo-R; the trpC terminator overlapped downstream of the targeted deletion genes was amplified with primers Neo-F/Down-check; the candidate genes for deletion was amplified with their corresponding primers. All information on the used primers is listed in Supplementary Table 2. Lane M, DL5000 DNA molecular marker.

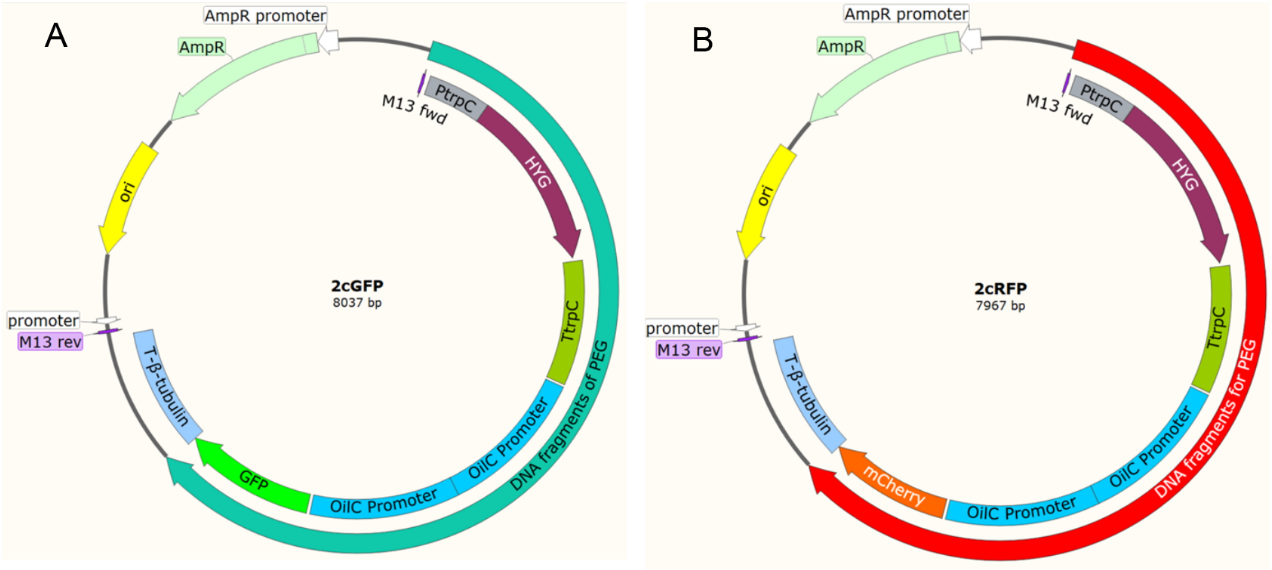

Supplementary Fig. 11 Maps of two fluorescent protein expression vectors. The green and mCherry fluorescent protein (GFP and mCherry) vectors contain hygromycin resistance genes and GFP (A) or mCherry (B) genes with 2×*Aspergillus nidulans* oliC promoters.

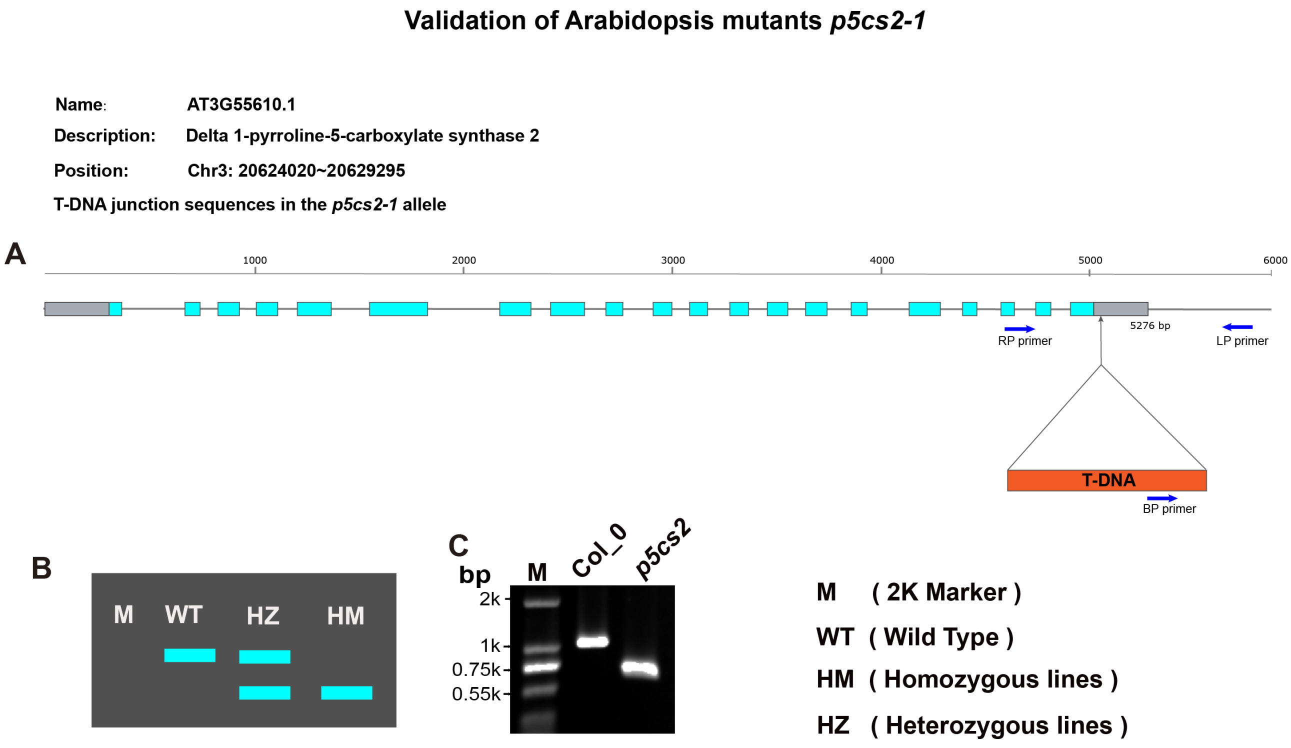

Supplementary Fig. 12 Confirmation of the *Arabidopsis p5cs2* mutants by PCR. (A) The framework of the T-DNA insertion site in *P5CS2* (At3g55610). The p5cs2-1 line (SALK_203144)

carries a single T-DNA insertion in intron 16. (B) The model for PCR screening of T-DNA insertions. HZ, HM, and WT represent hemizygous, homozygous, and wildtype. (C) PCR confirmation was performed using gene-specific primer pairs and combinations of either 5' or 3' gene-specific primers with T-DNA end-specific primers (Table S2). PCR results confirmed that *Arabidopsis p5cs2* mutant is homozygous line.

Supplementary Table 1 List of *S. sclerotiorum* strains used in the study

Strain	Strain abbreviation	Mycovirus[1]	Background strain	Fluorescent gene	Hyg[2]	Compatible with Ep-1PNA367[3]	Compatible with Sun-M	Reference
Ep-1PN	EP	SsDRV, SsRVL	Ep-1PN	—	S	Y	N	42,46
Ep-1PNA367	A367	—	Ep-1PN	—	S	Y	N	42
Ep-1PNA367GFP	A367G	—	Ep-1PN	GFP	R	Y	N	This study
1980	1980	—	1980	—	S	N	N	This study
1980mcherry	1980m	—	1980	mCherry	R	N	N	This study
1980GFP	1980G	—	1980	GFP	R	N	N	This study
1980R	1980R	—	1980	—	S	N	N	This study
Sun-MGFP9	SG9	—	Sun-M	GFP	R	N	Y	This study
SCH941A1	941A1	—	SCH941	—	S	N	N	81
SCH733	SCH733	SsNaV5	SCH733	—	S	Y	N	41
SZ-150	SZ150	SsHV1	SZ-150	—	S	N	N	42
SX276	SX276	SsEV1	SX276	—	S	Y	N	39
Ep-1PNA367T1	AT1	SsMYRV4	AT1	—	S	Y	N	52
WF-1	WF-1	SsPV1	WF-1	—	S	N	N	44
AH98	AH98	SsNSRV1	AH98	—	S	N	N	40

Mycovirus[1]: the full names of mycoviruses: SsDRV, Sclerotinia sclerotiorum debilitation-associated RNA virus; SsRVL, Sclerotinia sclerotiorum RNA virus L; SsNaV5，Sclerotinia sclerotiorum narnavirus 5; SsHV1, Sclerotinia sclerotiorum hypovirus 1; SsEV3, Sclerotinia sclerotiorum endornavirus 3; SsMYRV4, Sclerotinia sclerotiorum mycoreovirus 4; SsPV1, Sclerotinia sclerotiorum partitivirus 1; SsNSRV1, Sclerotinia sclerotiorum negative-stranded RNA virus 1

Hyg[2]: Hygromycin; R donates that the strain is resistant to hygromycin (50~300 μg/ml), and S indicates that the strain is susceptible to hygromycin (30 μg/ml).

Compatible with Ep-1PNA367[3]: Y donates that the strain is compatible with Ep-1PNA367, N donates that the strain is incompatible with Ep-1PNA367.

“−” : No mycovirus infection or no fluorescence gene label

Supplementary Table 2 The detailed information of primers used in the study.

Primer Name	Sequence(5'-3')	Mycovirus or gene
RT-PCR for mycoviruses detection		
SsDRV-F	AGCACACACTTCCAGATTCAACTCG	SsDRV
SsDRV-R	GAGACTTTGACTCATCCAACCATCG	
SsRVL-F	CCAGCCTTTCCGACACCGACT	SsRVL
SsRVL-R	GTCAGATTGGCGATAGTGGCGTAA	
SsHV1-F	GCGTAGACACAAGTTGCCTCAA	SsHV1
SsHV1-R	TGTTCCGTTGCCCTCATTCGTA	
SsEV3-F	GGGAAGGACTTACCCAATACG	SsEV3
SsEV3-R	GCTGTAACTCCTGTCTCGTAC	
SsMYRV4-F	CCAGTCGCCAAACTTCTTTATGATC	SsMYRV4
SsMYRV4-R	GACATTTCCCTTCGCATCAACTCA	
SsPV1-F	TTTCGTTATGGGCGACGACA	SsPV1
SsPV1-R	GGGACCATTTCTTGTCGGGT	
SsNSRV1-F	CCGTGGCTGGGACGCTTCAT	SsNSRV1
SsNSRV1-R	TGTCGTCCACCAGGCTTCTT	
Primers used for gene expression analysis of the candidate genes related to VIC		
Ssvic1-F	CCTTGGAAAGCATAGAGTGAGTTGGG	*Ssvic1* (sscle_03g027040)
Ssvic1-R	TCGACCCAAATACAGGCATGAAGAAT	
Ssvic2-F	ACAAAGTTGGTGCTTCCCCA	*Ssvic2* (sscle_16g107580)
Ssvic2-R	CGGGCTATTTGCCACCTAGT	
Ssvic3-F	TCCTGTCATTGCACCTGGTC	*Ssvic3* (sscle_16g01924)
Ssvic3-R	CAAGGGCCTGCCAGTAAAGA	
Ssvic4-F	TGTCGACGGTGTCAAAGAGG	*Ssvic4* (sscle_15g107070)
Ssvic4-R	CGTCCCGTCCCTTCTCAAAA	
Ssvic5-F	CGCCGTTATTGCCCATCTTG	*Ssvic5* (sscle_03g022180)
Ssvic5-R	CGCCCACCAATGTCTCTGAT	
Ssvic6-F	GCGTCAGCTAACCAAAGCAG	*Ssvic6* (sscle_08g064990)
Ssvic6-R	GGGCTCATTCGTTGTGCTTG	
Sscwr1-F	CGGAGTCGACACACTCAACA	*Sscwr1* (sscle_01g004270)
Sscwr1-R	ACATCCAAAGAATCGGCGGT	
Sscwr2-F	AAGAAAAGGCGACAGCTGGA	*Sscwr2* (sscle_02g014090)
Sscwr2-R	CGCAGCATTCCTTTGCACTT	
Sscwr3-F	AAGGGTGGCCTAACTGAAGC	*Sscwr3* (sscle_15g103830)
Sscwr3-R	CCCTCGTATCCGCTCACTTC	
Ssvib1-F	CCAAATTTCCGGATCTGTCACCAC	*Ssvib* (sscle_03g025080)
Ssvib1-R	TGTAGAAGCCGGAGAATGTGTCTGG	
Neo-F	GCTGCTCTGATGCCGCCGTGTT	Neomycin gene (G418)
Neo-R	GTTCTTCAGCAATATCACGGGTAGC	
Primers used for construction deletion vectors of the candidate genes related to VIC		
vic1-up-F	TTGCATTAACAATGGTTTGTTCAGGTCAA	upstream fragment of *Ssvic1*
vic1-down-R	TGTAGAAGCCGGAGAATGTGTCTGG	downstream fragment of *Ssvic1*
vic2-up-F	AGGATGGGTGTACATTTTAGGAGGG	upstream fragment of *Ssvic2*
vic2-down-R	CCCTGGATCTTGGCAGGCTAGTAG	downstream fragment of *Ssvic2*
vic3-up-F	CCTTCCATTCCTTTCTTCACTTAAGG	upstream fragment of *Ssvic3*
vic3-down-R	GGTACGCGCTCGCTTGGGAAACT	downstream fragment of *Ssvic3*
vic4-up-F	TACAGGATCAGCCGCAAGCCATT	upstream fragment of *Ssvic4*
vic4-down-R	GGCGCCTCACTTGATATATGTAAGGG	downstream fragment of *Ssvic4*
vic5-up-F	ACTGGGAAATAGAGGCTAGAGGGC	upstream fragment of *Ssvic5*
vic5-down-R	GGAGAGGGTTTCAGGAAGGCG	downstream fragment of *Ssvic5*
vic6-up-F	ATCTATTGGAAATAGGTTTAATAGCCC	upstream fragment of *Ssvic6*
vic6-down-R	AGATCAGAGGGAAATTCGAGGAGGG	downstream fragment of *Ssvic6*
cwr1-up-F	CCCCTGCAGACTTTCAATCATTCAATG	upstream fragment of *Sscwr1*
cwr1-down-R	TACATCACCAACTCGTCGATATGCC	downstream fragment of *Sscwr1*
cwr2-up-F	TGCGAGTAAAGCGCAAGCCAG	upstream fragment of *Sscwr2*

cwr2-down-R	TTCCAGACATCAAAAAATTCCGCCC	downstream fragment of *Sscwr2*
cwr3-up-F	CGAGGAATGGATGGCCAGAGGAA	upstream fragment of *Sscwr3*
cwr3-down-R	TTAACCAGCAAGGCACTTTGCC	downstream fragment of *Sscwr3*
vib1-up-F	TATCGCCGGGTTCGTATTCACATC	upstream fragment of *Ssvib1*
vib1-down-R	GGTGAATGTGGCGCGTCATTAGTAA	downstream fragment of *Ssvib1*
Primers used for gene expression of two proline biosynthesis precursor genes *p5cs1* and *p5cs2* in plants		
Ara_qP5CS1F	GAGCTAGATCGTTCACGTGCTTT	*p5cs1* in *Arabidopsis*
Ara_qP5CS1S	ACAACTGCTGTCCCAACCTTAAC	
Ara_qP5CS2F	GTTAAGCGTATCGTCGTCAAGGTT	*p5cs2* in *Arabidopsis*
Ara_qP5CS2S	CCTAAACGTCCAAGAGCCAATCT	
Ara_qACT2	GGTAACATTGTGCTCAGTGGTGG	actin in *Arabidopsis*
Ara_qACT2	AACGACCTTAATCTTCATGCTGC	
BnaA.P5CS1cF	GTGGGGATAAGCACAGGG	*p5cs1* in oilseed rape
BnaA.P5CS1cS	CCATCCTCTCCTAGTCTCC	
BnaA.P5CS2bF	GATGCACATTTGTGGGCTAGA	*p5cs2* in oilseed rape
BnaA.P5CS2bS	GCTTCTAGAGCGTTGGCTATATCT	
BnaC.UBQ11F	GTTGATCTTCGCTGGAAAAC	*actin* in oilseed rape
BnaC.UBQ11S	CCATTAAAGACGGCTCGATG	
P5CS2-LP	ATGGTTTGTCTGTGAACCGTC	*p5cs2* and T-DNA confirmation of *Arabidopsis* mutant
P5CS2-RP	AATGGCCAATGTGATTTGTTC	
LBb1.3	ATTTTGCCGATTTCGGAAC	T-DNA confirmation of *Arabidopsis* mutant
Primers used for the expression detection of genes related to G proteins and ROS		
sscle_05g042960R	CTTCCATCATTCTGTTCTTG	SsGα1 (sscle_05g042960)
sscle_05g042960F	CATCACCACCTTCGTAAT	
sscle_07g056270F	ATATCAACGCCATCCAAT	SsGβ1 (sscle_07g056270)
sscle_07g056270R	TCTATCAGCACGAATGTC	
sscle_06g053750F	ATCATCCAATACACCAGTC	SsGγ (sscle_06g053750)
sscle_06g053750R	ATCACCAAACAACATCCA	
sscle_03g023580F	ACTTGAACGAGTCTAAGC	SsGβ2 (sscle_03g023580)
sscle_03g023580R	AACCTTGCTCTTCTTCTC	
sscle_11g084840F	GGATTCAGCACCATACTT	SsGα2 (sscle_11g084840)
sscle_11g084840R	CTCGTAGATACCAGTTGTC	
sscle_16g109000F	GGCAAGTCAAGTTGTTAC	SsGα3 (sscle_16g109000)
sscle_16g109000R	CCTCTACTGTCATTCTCAAT	
SsActin-F	CTTCCATTGTCGGTCGTCCC	*actin* in *S. sclerotiorum*
SsActin-R	TGACGACACCGTGCATTGGG	
Ssnox1F	CTGCTCATGCCAAGATTAGA	*Ssnox1* (sscle_09g069850)
Ssnox1R	CCAACGCCATCCTTCATAT	
Ssnox2F	CTGCTCATGCCAAGATTAGA	*Ssnox2* (sscle_14g099710)
Ssnox2R	CCAACGCCATCCTTCATAT	
